# Arithmetic mismatch negativity and numerical magnitude processing in number matching

**DOI:** 10.1186/1471-2202-12-83

**Published:** 2011-08-11

**Authors:** Yi-Fang Hsu, Dénes Szücs

**Affiliations:** 1 Centre for Neuroscience in Education, Department of Experimental Psychology, University of Cambridge; Downing site, Cambridge CB2 3EB, UK

## Abstract

**Background:**

This study examined the relationship of the arithmetic mismatch negativity (AMN) and the semantic evaluation of numerical magnitude. The first question was whether the AMN was sensitive to the incongruity in numerical information per se, or rather, to the violation of strategic expectations. The second question was whether the numerical distance effect could appear independently of the AMN. Event-related potentials (ERPs) were recorded while participants decided whether two digits were matching or non-matching in terms of physical similarity.

**Results:**

The AMN was enhanced in matching trials presented infrequently relative to non-matching trials presented frequently. The numerical distance effect was found over posterior sites during a 92 ms long interval (236-328 ms) but appeared independently of the AMN.

**Conclusions:**

It was not the incongruity in numerical information per se, but rather, the violation of strategic expectations that elicited the AMN. The numerical distance effect might only temporally coincide with the AMN and did not form an inherent part of it.

## Background

Numerous event-related potential (ERP) studies demonstrated that arithmetic mismatch in addition/multiplication verification tasks and number matching tasks elicits a negative-going potential [1-9]. Here we call this phenomenon arithmetic mismatch negativity (AMN). Some properties of the AMN remained unresolved in previous studies. First, it is unclear whether the AMN is elicited by the incongruity in numerical information per se, or rather, by the violation of strategic expectations in a certain experimental paradigm. Second, it has been shown that the ERP amplitude in the time window of the AMN is sensitive to semantic manipulations [2,10,11]. However, it is unknown whether these semantic amplitude effects are inherent to the AMN itself, or rather, they only coincide with it. This study set out to respond to the above two questions.

Typically, the AMN is demonstrated in arithmetic verification tasks and number matching tasks. In arithmetic verification tasks, participants see equations consisting of operands and operators (e.g. 3 × 4; 3+4) followed by correct or incorrect arithmetic outcomes [1-5,9]. The AMN appears as incorrect arithmetic outcomes elicit more negative-going ERPs than correct arithmetic outcomes. In number matching tasks, participants see pairs of numbers presented serially which are either matching or non-matching to each other [8]. The AMN appears as non-matching numbers elicit more negative-going ERPs than matching numbers. In previous studies, the AMN as a negativity emerging between 250-450 ms was referred to by various names, e.g. N270, N400, and N300 [1-4,9,12]. However, here we use the neutral term "AMN" considering that the functional nature of this effect has not yet been determined.

One question is whether the AMN is sensitive to the incongruity in numerical information per se, or rather, to the violation of strategic expectations when participants encounter unexpected stimuli within the context of arithmetic tasks. The former possibility would suggest that the AMN is a specific signal in number processing. The latter possibility would suggest that the AMN is an ERP effect reflecting general mismatch detection, similar to the negative-going ERPs reported in non-arithmetic tasks, such as colour matching task [8], shape matching task [13], and category matching task [6].

Another issue is that the semantic content of stimuli seems to influence the ERP amplitude in the time window of the AMN. For example, Niedeggen and Rösler [2] documented that the amplitude of the AMN is modulated as a function of numerical distance between presumed and perceived arithmetic outcomes. However, it is an open question whether semantic relations influenced the AMN per se. In fact, some studies focusing on semantic relations in arithmetic found the numerical distance effect in the absence of the AMN [10,11]. This suggests that the numerical distance effect might only temporally coincide with the AMN.

The dissociation of arithmetic incongruence, general mismatch detection, and semantic effects potentially overlapping with the AMN in simple numerical tasks is not straightforward. This is so because incorrect arithmetic outcomes are probably associated with strong and subjective "mismatch" even when they are frequent. For example, the AMN appears in response to incorrect arithmetic outcomes even when they are presented in 80% of the trials [5]. One way around this problem is to use a number matching task which not only elicits the AMN but also elicits the semantic analysis of numerical magnitude [14-16]. In such a task participants decide whether pairs of numbers are matching or non-matching to each other in terms of physical similarity. This allows for independent manipulation of arithmetic incongruence and general mismatch detection. Specifically, arithmetic incongruence and general mismatch detection can be separated by making physically non-matching numbers appear frequently and physically matching numbers appear infrequently. If the AMN is sensitive to the incongruity in numerical information per se, it should be found in frequent non-matching trials. In contrast, if the AMN is sensitive to the violation of strategic expectations, it should be found in infrequent matching trials. The second question concerns whether the amplitude modulations as a function of numerical distance are inherent to the AMN, or rather, they reflect a process which temporally coincides with the AMN. With regard to this question, we expected on the basis of previous studies [10,11] that the amplitude modulations of numerical distance may be seen in ERPs even in the absence of the AMN.

## Methods

### Participants

16 adults (average age 26; 6 males; 3 left-handed) were recruited from the University of Cambridge and surrounding community. All participants had normal or corrected-to-normal vision and were neurologically healthy as indicated by a self-report. Participants gave written informed consent and were paid for participation. Procedures were approved by the Cambridge Psychology Research Ethics Committee of the University of Cambridge.

Each participant's general ability was assessed using two subtests (Vocabulary and Block Design) of the Wechsler Abbreviated Scales of Intelligence (WASI). Their mathematics achievement was measured using the Math Computation subtest of the Wide Range Achievement Test 4 (WRAT4). Participants' scores in tests of general ability and mathematics achievement were within normal range. Standardised scores in the Vocabulary and Block Design subtest of the WASI were within the range of 52-73 and 55-69, respectively. Standardised scores in the Math Computation subtest of the WRAT4 were within the range of 100-136.

### Stimuli and procedures

4 digits (1, 2, 8, 9) were paired to create 12 stimuli (1-1, 2-2, 8-8, 9-9, 1-2, 2-1, 8-9, 9-8, 1-8, 8-1, 2-9, 9-2). The digits assigned to each condition were completely balanced. Each stimulus was presented 20 times so that one-third of the trials (80 trials) contained physically matching digits and two-thirds of the trials (160 trials) contained physically non-matching digits. In the non-matching trials, half of the trials contained digits that differed by a numerical distance of 1 (small numerical distance) and half of the trials contained digits that differed by a numerical distance of 7 (large numerical distance). Stimuli were presented on a 17-inch computer screen, white on a black background in Times New Roman (size 40) font. The Presentation software package (Neurobehavioral Systems, Inc.) was used to control the presentation of the stimuli.

The experiment consisted of 2 blocks. Each block contained 120 trials (40 matching trials, 40 non-matching trials of small numerical distance, and 40 non-matching trials of large numerical distance). A trial started with a fixation sign (a drawing of an eye) presented for 500 ms. The screen then remained blank for 1000 ms. This was followed by the presentation of the stimulus for 1000 ms. Participants were required to press one button if the digits were matching and another button if the digits were non-matching. The assignment of response hands was counterbalanced across participants. The next trial started after a random intertrial interval of 300-400 ms after the response or the offset of the stimulus. Stimuli were presented in pseudo-random order with the constraint that the same stimuli did not appear in consecutive trials. The experiment was preceded by 24 practice trials.

### Data analysis

Repeated-measures ANOVA were conducted on behavioural data in matching trials, non-matching trials of small numerical distance, and non-matching trials of large numerical distance. Planned comparisons between non-matching trials of small numerical distance and non-matching trials of large numerical distance were made with 2-tailed paired-samples t tests.

EEG was recorded with 65-channel sensor nets (Electrical Geodesics, Inc.). The sampling rate was 500 Hz and an online bandpass filter of 0.01-70 Hz was used. The data was bandpass filtered between 0.3-30 Hz offline and was recomputed to average reference. Epochs extended from -100 ms to 600 ms relative to the presentation of stimulus, using a 100 ms pre-stimulus baseline. Epochs containing voltage deviations exceeding +/-100 μV relative to baseline at any of the recording electrodes were rejected.

For the examination of the AMN, repeated-measures ANOVA were conducted on the amplitude of the ERPs averaged across the time window of the AMN (240-300 ms) in matching trials, non-matching trials of small numerical distance, and non-matching trials of large numerical distance. The AMN was considered significant when both the main effect reached significance (p < 0.05) and the Bonferroni-corrected pairwise comparisons between matching trials and non-matching trials of small/large numerical distance reached significance (p < 0.017) on at least 3 electrodes. For the examination of the numerical distance effect, point-by-point t-tests were conducted comparing the amplitude of the ERPs in non-matching trials of small numerical distance and non-matching trials of large numerical distance. The numerical distance effect was considered significant when the main effect reached significance (p < 0.05) on at least 3 electrodes across at least 20 consecutive samples.

## Results

Table 1 summarises the means and standard deviations of behavioural data. Repeated-measures ANOVA revealed a significant main effect of condition in response accuracy (F(2,30) = 5.42, p < 0.05) and a marginal main effect of condition in RT (F(2,30) = 2.98, p < 0.1). Planned comparisons revealed that participants responded less accurately (t(15) = -2.30, p < 0.05) and more slowly (t(15) = 2.23, p < 0.05) to small numerical distance trials than to large numerical distance trials.

**Table 1:** The means and standard deviations of behavioural data

	Response accuracy (%)		RT (ms)	
	**M**	**SD**	**M**	**SD**

Matching	95.23	5.21	525.78	60.56

Non-Matching: Small numerical distance	97.58	2.01	518.02	65.46

Non-matching: Large numerical distance	98.44	2.30	511.25	70.02

**Figure 1:**
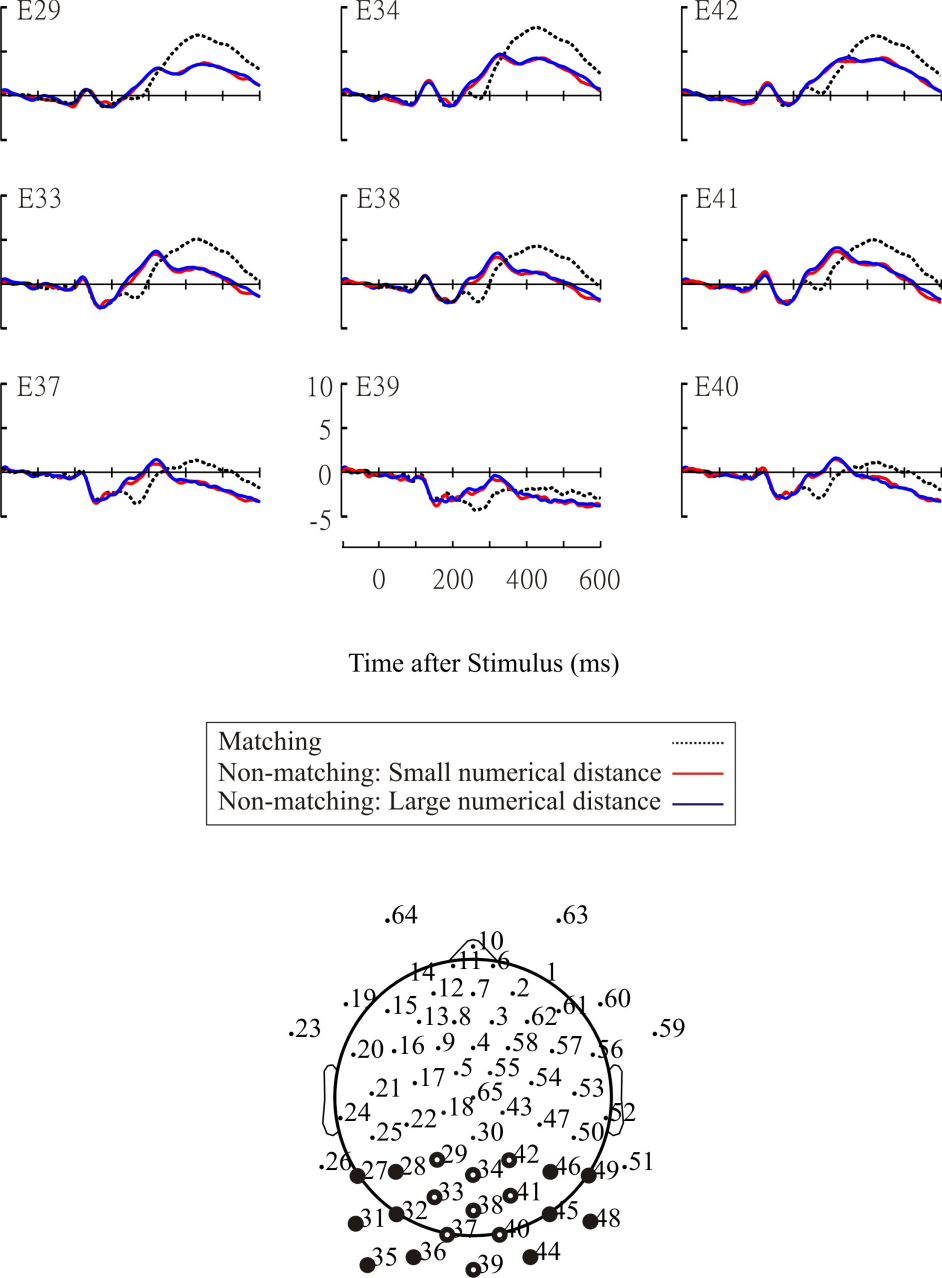
**The AMN**. Marked dots in the montage represent the locations of electrodes showing the AMN. Representative electrodes are marked as white while other electrodes are marked as black.

**Figure 2:**
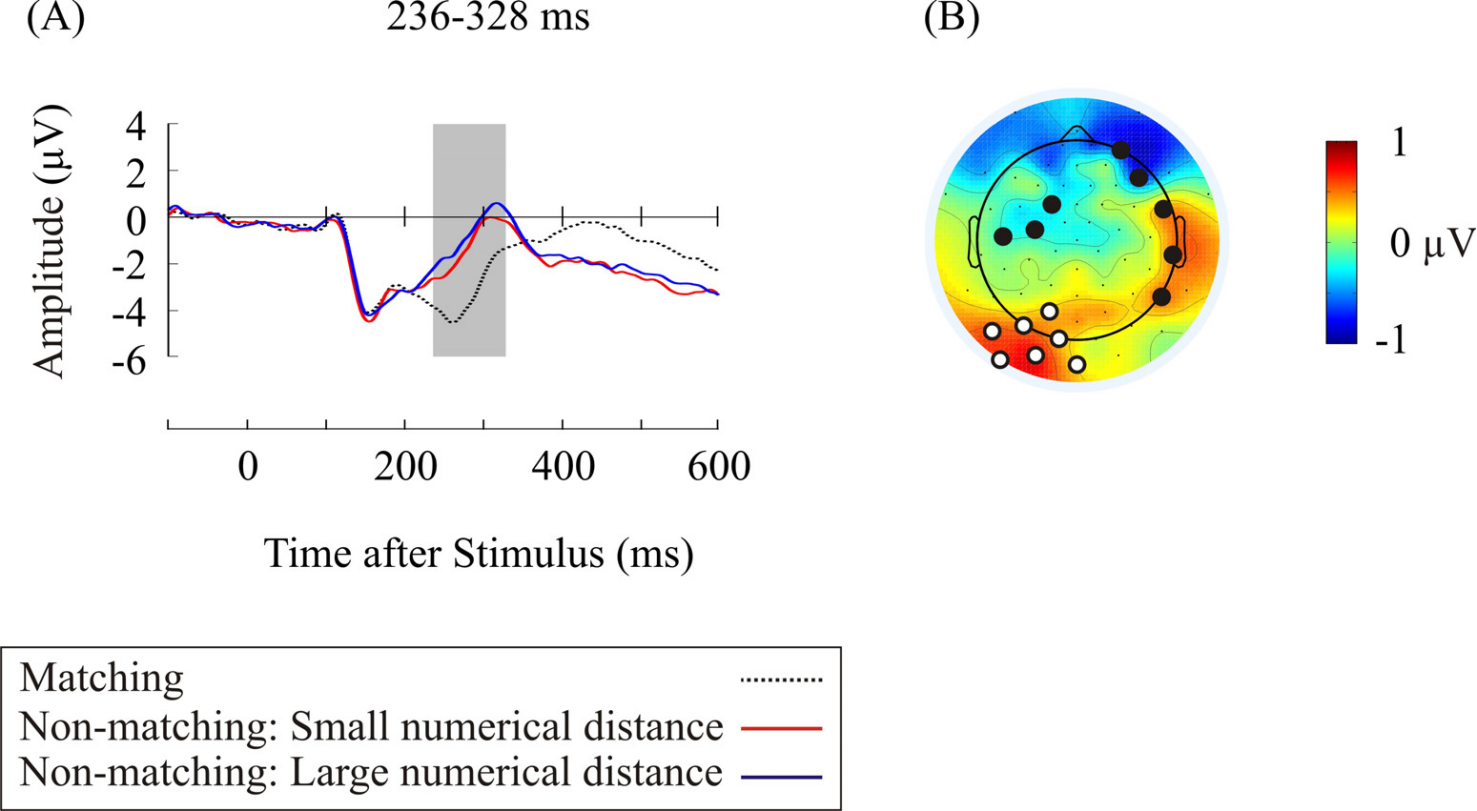
**The numerical distance effect**. (A) ERPs averaged across representative electrodes over posterior sites (Electrode 31, 32, 33, 35, 36, 37, 39) showing the numerical distance effect. Shaded area represents the time interval showing the numerical distance effect. (B) Topographic distribution of difference ERPs (large numerical distance condition minus small numerical distance condition) during the time interval showing the numerical distance effect. Marked dots in the topographic map represent the locations of electrodes showing the numerical distance effect. Representative electrodes are marked as white while other electrodes are marked as black.

Figure 1 shows the AMN at representative electrodes. The significant main effect of condition was found over posterior sites (Table 2). The AMN was enhanced in matching trials relative to non-matching trials of small/large numerical distance. Figure 2 shows the time course and the topographic distribution of the numerical distance effect. The amplitude of the ERPs was significantly less positive in non-matching trials of small numerical distance than in non-matching trials of large numerical distance over posterior sites during a 92 ms long interval (236-328 ms).

**Table 2:** List of electrodes showing the AMN

Electrode	ANOVA		Pairwise comparison: Matching vs Non-matching of small numerical distance	Pairwise comparison: Matching vs Non-matching of large numerical distance
27	F(2,30) = 15.23	p < 0.001	p = 0.002	p = 0.001
28	F(2,30) = 18.48	p < 0.001	p < 0.001	p = 0.001
29	F(2,30) = 9.28	p = 0.001	p = 0.003	p = 0.007
31	F(2,30) = 25.40	p < 0.001	p = 0.001	p < 0.001
32	F(2,30) = 32.41	p < 0.001	p < 0.001	p < 0.001
33	F(2,30) = 21.84	p < 0.001	p < 0.001	p < 0.001
34	F(2,30) = 12.61	p < 0.001	p = 0.003	p = 0.001
35	F(2,30) = 11.42	p < 0.001	p = 0.013	p = 0.001
36	F(2,30) = 19.53	p < 0.001	p = 0.003	p < 0.001
37	F(2,30) = 19.60	p < 0.001	p = 0.001	p < 0.001
38	F(2,30) = 23.67	p < 0.001	p < 0.001	p < 0.001
39	F(2,30) = 23.12	p < 0.001	p < 0.001	p < 0.001
40	F(2,30) = 22.48	p < 0.001	p < 0.001	p < 0.001
41	F(2,30) = 18.14	p < 0.001	p = 0.002	p < 0.001
42	F(2,30) = 9.68	p = 0.001	p = 0.007	p = 0.004
44	F(2,30) = 17.29	p < 0.001	p = 0.002	p < 0.001
45	F(2,30) = 27.61	p < 0.001	p < 0.001	p < 0.001
46	F(2,30) = 11.68	p < 0.001	p = 0.005	p = 0.001
48	F(2,30) = 18.20	p < 0.001	p = 0.001	p < 0.001
49	F(2,30) = 20.30	p < 0.001	p = 0.001	p < 0.001

## Discussion

We used the number matching task to address two questions. First, we examined whether the AMN was sensitive to the incongruity in numerical information per se, or rather, to the violation of strategic expectations. Second, we examined whether the numerical distance effect formed an inherent property of the AMN, or rather, they reflected a process which temporally coincided with the AMN.

In response to our first question, we found that the AMN appeared in infrequent matching trials relative to frequent non-matching trials over posterior sites. The presence of the AMN in infrequent trials irrespective of their numerical matching property suggests that it is not the incongruity in numerical information per se, but rather, the mismatch within the context of arithmetic tasks that elicits the AMN. Therefore, we suggest that the AMN is an ERP effect reflecting the detection of mismatch in relation to the violation of strategic expectations [6,8,13]. In other words, the AMN can probably be triggered whenever a mismatch is encountered as proposed by the theory of Wang and colleagues [8,13]. Our results are in coherence with the only study that systematically investigated the effect of stimulus probability on the AMN in the arithmetic verification task [5]. In that study, the amplitude of the AMN became smaller on parietal electrodes with increasing probability of incorrect arithmetic outcomes. However, the AMN to incorrect arithmetic outcomes remained conspicuous even when their probability reached 80%. This can be explained by the influence of strategic expectations in the arithmetic verification task: Participants tend to expect correct arithmetic outcomes even when their probability is very low. In contrast, in the number matching task used here, the influence of strategic expectations inherent to the task could be more successfully dissociated.

In response to our second question, the numerical distance effect demonstrated in the behavioural data suggests that semantic analysis of numerical magnitude occurred in this task as expected on the basis of previous studies [14-16]. In line with our hypothesis, the numerical distance effect appeared independently of the AMN in frequently presented non-matching trials. Furthermore, the numerical distance effect temporally coincided with the AMN. This finding suggests that the numerical distance effect is not necessarily linked to the AMN, but rather, it might reflect a numerical magnitude evaluation process which temporally coincides with the AMN. The data is in good agreement with previous studies. First, the pattern of the numerical distance effect is similar to previous studies in that the small numerical distance condition, relative to the large numerical distance condition, triggered enhanced negativity in the ERPs [12]. Second, the results are in accordance with previous studies reporting semantic effects on the ERPs even in the absence of the AMN [10,11]. It is important to note that we used exactly the same stimuli in both small numerical distance condition and large numerical distance condition. Therefore, the difference in the ERPs between different numerical distance conditions cannot be attributed to perceptual factors. The numerical distance effect in the time window of the AMN is probably the electrophysiological correlate of the behavioural numerical distance effect related to the automatic processing of symbolic numerical magnitude [14-16].

## Conclusions

In this ERP study, we found that the AMN is an ERP effect reflecting general mismatch detection, that is, the violation of strategic expectations. Furthermore, the numerical distance effect was not necessarily linked to the AMN. Instead, it might reflect a numerical magnitude evaluation process which temporally coincides with the AMN.

## Authors' contributions

Both authors designed the study, contributed to the preparation of the manuscript, and approved the final draft.
